# Elucidation of putative binding partners for the protein encoded by ORF149 of cyprinid herpesvirus 3 in goldfish (*Carassius auratus*)

**DOI:** 10.1111/jfd.13171

**Published:** 2020-04-22

**Authors:** Simon Menanteau–Ledouble, Michael Gotesman, Ebrahim Razzazi‐Fazeli, Sven M. Bergmann, Mansour El‐Matbouli

**Affiliations:** ^1^ Clinical Division of Fish Medicine Department for Farm Animals and Veterinary Public Health University of Veterinary Medicine Vienna Austria; ^2^ Department of Biology New York City College of Technology of the City University of New York Brooklyn NY USA; ^3^ Protein Division Ibex Biosciences LLC Cumberland MD USA; ^4^ VetCore Facility for Research University of Veterinary Medicine Vienna Vienna Austria; ^5^ Federal Research Institute for Animal Health Friedrich‐Loeffler Institut Greifswald‐Insel Riems Germany

**Keywords:** antibody, cytoskeletal, LC‐MS/MS, mitochondria, protein purification

Cyprinid herpesvirus 3 (CyHV‐3) was identified by Bretzinger, Fischer‐Scherl, Oumouma, Hoffmann, and Truyen (1999) and Hedrick et al. (2000) as the aetiological agent of a viral disease termed KHVD, which can cause mortality as high as 80%–100% in common carp *Cyprinus carpio* (Reviewed in Gotesman, Kattlun, Bergmann, & El‐Matbouli, 2013). CyHV‐3 is a double‐stranded DNA virus consisting of a 295 kB genome encoding164 putative open reading frames (ORFs), and mass spectrometry analysis of viral particles has identified 40 proteins packaged in a mature virion including 22 structural, 3 capsid, 2 tegument and 13 envelope proteins (Michel, Leroy, et al., 2010). Furthermore, the immunogenic and vaccine potentials of several epitopes of CyHV‐3 have been investigated, including Orf12 (Kattlun, Menanteau‐Ledouble, & El‐Matbouli, 2014) which is readily recognized by the immune system of carp and Orf81 for which conflicting evidence exists (Kattlun et al., 2016; Zhou et al., 2014), although little such research has been conducted in goldfish (*Carassius auratus*). In previous reports, our group elucidated pathogen–host interactions in CyHV‐3‐infected *C. auratus* through the use of monoclonal antibody‐linked pulldown assay followed by electro‐spray ionization mass spectrometry (ESI‐MS) as described in Gotesman, Menanteau‐Ledouble, and El‐Matbouli (2016). *C. auratus* is a non‐symptomatic carrier of CyHV‐3, and previous studies demonstrated that in *C. auratus*, several host defence proteins interact with CyHV‐3 (Bergmann et al., 2010; Gotesman, Abd‐Elfattah, Kattlun, Soliman, & El‐Matbouli, 2014). Interestingly, several of these proteins were not found to interact in the common carp, the susceptible carp host for CyHV‐3 (Gotesman, Soliman, & El‐Matbouli, 2013). A recent study by Torrent et al. (2016) has demonstrated that the IgMs of asymptotic CyHV‐3 surviving carp recognize an epitope derived from the amino‐terminal of the glycoprotein coded by the ORF149 of CyHV‐3 (Orf149). Monoclonal antibodies (mAbs) were generated by immunizing mice with purified CyHV‐3 particles (Cabon et al., 2017), and these mAbs were used to detect the virus in common carp brain cells by enzyme‐linked immunosorbent assay (Bergmann et al., 2017).

Pulldown assays use antibodies to capture a “bait” protein in an affinity resin, and in the present study, we applied a pulldown assay to investigate the proteins interacting with the Orf149 epitope: Orf149 mAbs were linked to N‐hydroxysuccinimide (NHS)‐activated agarose columns (Gotesman et al., 2016) to capture and identify host proteins interacting with CyHV‐3. Kidney samples of *C. auratus* previously infected by intraperitoneal injection with 200 µl of CyHV‐3 virus at a concentration of 10^4^ TCID_50_ (Kattlun et al., 2014, 2016) were lysed using a Tissue Lyser (Qiagen). The sample were resuspended in non‐denaturing buffer (Gotesman et al., 2014; Gotesman, Soliman, et al., 2013) and passed through the columns by gravity filtration to expose the extracted host proteins to the agarose‐linked mAbs. The columns were rinsed 8 times with phosphate‐buffered saline (PBS) to ensure that unbound extracts were washed away as measured by spectrophotometry_280_ (OD = 0) and the bound proteins were eluted from the column using glycine (pH 3) into microcentrifuge tubes containing neutralizing Tris base (pH 8). The entire eluted fraction was analysed by liquid chromatography tandem mass spectrometry LC‐MS/MS analysis (performed at the VetMedUni VetCore facilities) to elucidate host proteins that putatively interact with Orf149.

The majority of the proteins identified (Table 1A) were identical to proteins previously identified in *C. carpio* (Gotesman, Soliman, et al., 2013) as well as using a different epitope of CyHV3 species (Gotesman et al., 2014), including cytoskeletal, elongation factors and enzymatic proteins (Table 1A). The cytoskeletal protein actin (Sandquist, Kita, & Bement, 2011), which was detected in both CyHV‐3‐positive and CyHV‐3‐negative samples, serves as a track for both conventional and unconventional myosins (Moen, Johnsrud, Thomas, & Titus, 2011) and plays a role in intracellular translocation and cell remodelling (Gotesman, Hosein, & Gavin, 2010, 2011).

**Table 1:** (A) Proteins identified that were overlapping from previous studies (Gotesman et al., 2014; Gotesman, Soliman, et al., 2013). (B) Proteins identified in both the positive and negative samples that were non‐overlapping from previous studies. (C) Unique proteins identified in this study from CyHV‐3‐positive samples

Uniprot Ref. #		Role	Coverage [%]	# Peptides	MW [kDa]	calc. pI
(A) Overlapping from previous studies (Gotesman et al., 2014; Gotesman, Soliman, et al., 2013)						
P53479	Actin, alpha skeletal muscle	Cytoskeletal protein	12	4	41.9	5.39
P83750	Actin, cytoplasmic 1	Cytoskeletal protein	12	4	41.7	5.48
Q800W9	Elongation factor 1‐alpha	Transcription Factor	4	2	50	9.09
M9T843	Haemoglobin alpha	Circulatory protein	13	3	15.6	8.85
Q9DGE4	Myeloid protein‐1	Circulatory protein	19	2	17.5	9.55
(B) Non‐overlapping from previous studies (Gotesman et al., 2014; Gotesman, Soliman, et al., 2013)						
O42326	Metalloendopeptidase	Enzymatic	7	2	31.3	9.42
(C) Unique to CyHV‐3 infected samples						
X4Z1X5	Mitochondrial cytochrome c	Mitochondrial	28	3	11.5	9.54

Another protein identified was the eukaryotic elongation factor 1 alpha (eEF1A) which has a diverse set of functions in the cell including interactions with the cytoskeleton (reviewed in Sasikumar, Perez, & Kinzy, 2012). Interestingly, certain RNA viruses interact with eF1A directly to aid in viral replication (Sasikumar et al., 2012) and this could explain the recovery of cytoskeletal protein actin by the pulldown assay. Because eEF1A’s activity is hijacked for viral propagation, it is plausible that an antibody targeting the glycoprotein Orf149 that interacts with the cell membrane of the host protein would also detect this cytoskeletal protein. This explanation is further supported by the fact that eEF1A was also pulled down by this assay.

Myeloid protein 1 is the final member of previously identified proteins. It is no surprise that a haemoglobin protein was recovered in the CyHV‐3‐positive samples (Table 1A) because CyHV‐3 is detectable in various regions of the circulatory system (Reviewed by Michel, Fournier, Lieffrig, Costes, & Vanderplasschen, 2010) including the hematopoietic tissue in the spleen (Lee et al., 2016).

The results from this trial also suggested that the mAb was able to capture the same metalloendopeptidase (metalloendopeptidase 042326) in both the infected and non‐infected samples (Table 1B). More importantly, a unique, mitochondrial cytochrome c protein (mitochondrial cytochrome C X4Z1X5) was also detected in the CyHV‐3‐positive samples. Intriguingly, this protein had not been previously implicated in CyHV‐3 infections (Table 1C). In our previous study, we identified interactions of CyHV‐3 with mitochondrial enzymes involved in ATP synthesis (Gotesman et al., 2014; Gotesman, Soliman, et al., 2013), and in this study, another mitochondrial protein was shown to interact with CyHV‐3. Cytochrome c‐like metalloendopeptidase is known to coordinate with metal ions for correct functions and has implications in immune function and disease (Bond & Jiang, 1997), and it is interesting to speculate what role the mitochondria plays in CyHV‐3 infection. Whether CyHV‐3 alters the activity of the mitochondrial machinery to produce higher amounts of ATP (Murata et al., 2000) or modulates apoptosis factors (Cotter & Blaho, 2009) to either increase viral replication (Aubert, Pomeranz, & Blaho, 2007; Zhou & Roizman, 2000) or the release of mature viruses via apoptosis (Zhang, Tang, & Xu, 2014), respectively, remains unclear. Interestingly, some viruses such as spring viremia of carp virus (SVCV) are known to modulate ROS (reactive oxidative species) production (Liu et al., 2017; Shao et al., 2016). Antimycin A (a small molecule inhibitor of cellular respiration) is known to inhibit the mitochondrial complex III, reducing ROS production in SVCV‐infected cells and inhibiting the transcription of SVCV glycoprotein and viral replication (Zhao et al., 2018). Alternatively, CyHV‐3 may curtail the production or release of ROS by the mitochondria to reduce the cell's natural viral defence mechanism (Gonzalez‐Dosal, Horan, & Paludan, 2012; Gonzalez‐Dosal et al., 2011). Such viral strategies have been previously reported, for example, among the important viral diseases that affect domesticated poultry, the fusogenically activated F and HN glycoproteins of Newcastle disease perturb mitochondrial fusion/fission haemostasis (Ren et al., 2019). The fact that CyHV‐3 can putatively interact with this aforementioned enzyme and other mitochondrial components raises interesting questions regarding how this virus modulates the natural host immune response and mitochondria for increased viability.

Pulldown assays have demonstrated good specificity in the past, and, because of the extensive cleaning steps, it is unlikely that our assay would have detected proteins that did not interact with the Orf149 protein. Indeed, using a different bait protein resulted in a different set of purified proteins. This confirmed that interactions between bait and prey proteins were critical in the purification process. In future research, it would be interesting to further investigate the interactions of CyHV‐3 with host proteins in different species, for example using other immunoprecipitation methods such as co‐immunoprecipitation.

The aquamedicine field is rapidly adapting unconventional approaches for the detection, characterization and treatment of emergent threats to marine and aquaculture industries (Reviewed by Gotesman, Menanteau‐Ledouble, Saleh, Bergmann, & El‐Matbouli, 2018). Interactions with host cells are one of the most critical aspects of viral infections; therefore, such studies can greatly improve our understanding of the disease. Moreover, such studies could potentially suggest new therapeutic possibilities.

## CONFLICT OF INTEREST

None.

## Data Availability

The data sets used and/or analysed during the current study are available from the corresponding author on reasonable request.
